# Internal Medicine Residency Program in Guyana: A Collaborative Model for Sustainable Graduate Medical Education in Resource-Limited Settings

**DOI:** 10.3389/fpubh.2017.00112

**Published:** 2017-05-29

**Authors:** Dev Persaud, Joanna Cole, Ramdeo Jainarine, Zahira Khalid

**Affiliations:** ^1^Internal Medicine Residency Program, Georgetown Public Hospital Corporation, Georgetown, Guyana; ^2^Infectious Disease, Veteran Affairs, Spokane, WA, United States; ^3^McMaster University, Hamilton, ON, Canada

**Keywords:** global health, Medical Education, Collaborations, Collaborative Learning, Collaborative Research, Internal Medicine, residency training, residency program

## Abstract

The Georgetown Public Hospital Corporation (GPHC) started the Internal Medicine/Infectious Diseases residency program in 2013. It was a collaborative initiative between GPHC and University of Maryland. Since that time the program has gone through many trials and developed new partnerships and collaboration and emerged as a young successful program with close international links that have worked and persevered in developing the successful academic and professional careers of its residents. International collaborations have resulted in applying innovative methods of teaching to deliver the curriculum in a sustainable manner in a resource-limited setting. The article discusses in detail the history of the program and the roles that the collaborative partners have played in the evolution of the program.

## A Little about Guyana

**Figure 1:**
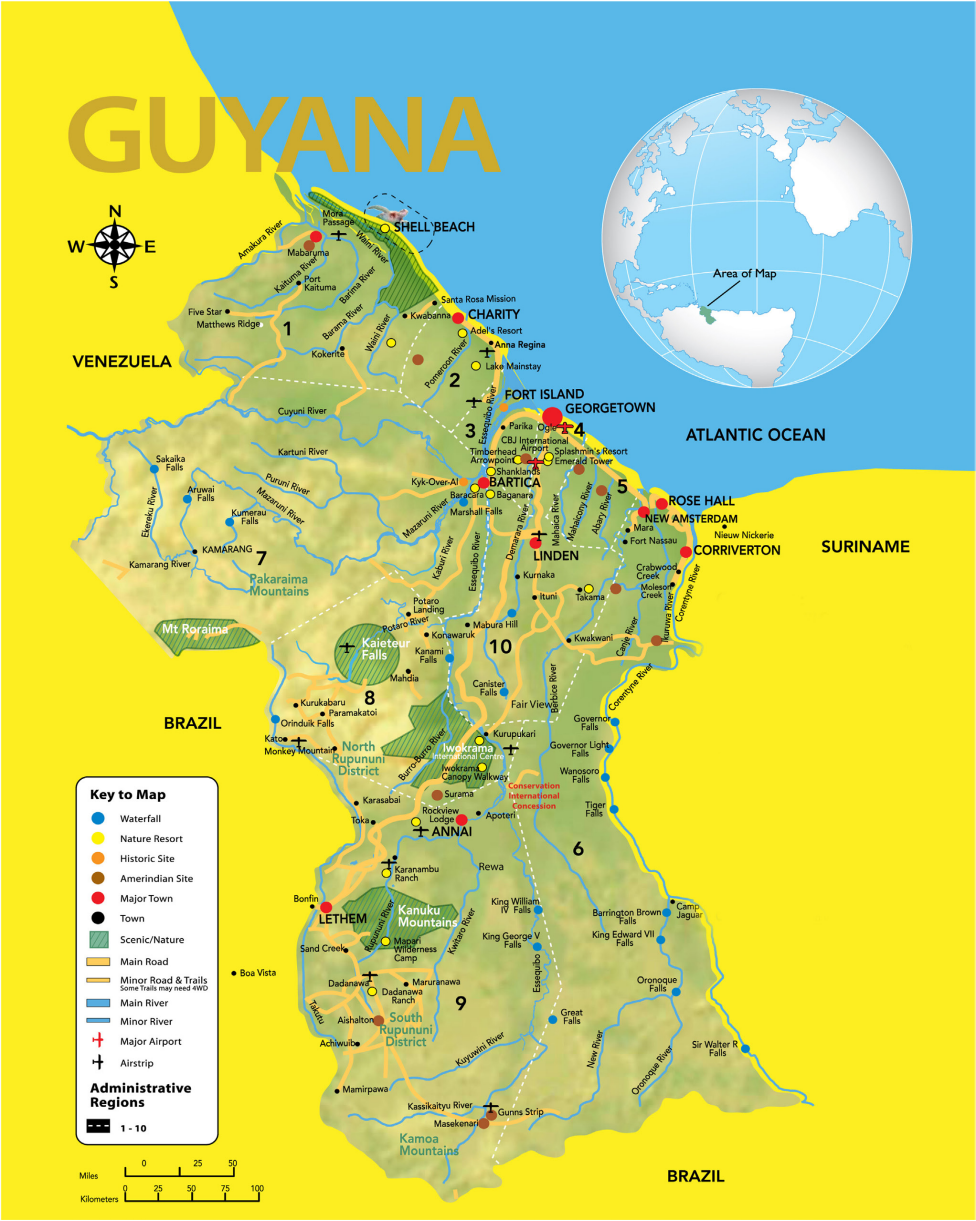
**Map of Guyana**.

The Cooperative Republic of Guyana [(Figure 1), (1)] is a small country on the northeastern coast of South America. Its population of approximately 750,000 is multiethnic and multicultural, with 43% Indo-Guyanese, 30% Afro-Guyanese, 17% mixed and the Indigenous Peoples, known as Amerindians, contribute to less than 10% of the populace. In contrast to most of its South American counterparts, where the overwhelming majority of the population lives in large cities with the average urban population for the Continent being close to 80% (2), presently only one in four Guyanese lives in a city or town (3). This difference being largely due to the lack of industrialization in urban centers in Guyana (4).

Over the past five decades, Guyana’s population annual growth rate has fluctuated widely due to the influence of multiple factors, such as the political establishment and socioeconomic conditions of the day, on the degree of outward migration. For example, during the 1980s when Guyana’s economy became heavily indebted and its currency was significantly devalued, the population declined to its lowest in modern times, due to an increased rate of emigration (5). Differences in earnings among countries and the availability of the labor market in other Caricom member states are also certainly factors that encourage migration (6). The largest numbers migrate from urban areas and include many well-educated professionals. In fact, Guyana has the highest rate of emigration of individuals with tertiary education in the world, where nine out of every 10 tertiary educated persons are migrating (7).

## The Need for In-Country Graduate Medical Education Programs

Like many of its Caribbean counterparts, Guyana is experiencing an epidemiologic transition with the highest burden of morbidity and mortality now being due to non-communicable diseases, such as ischemic heart disease and diabetes mellitus, while some communicable diseases still present formidable challenges. Human immunodeficiency virus (HIV) infection/AIDS is also a significant contributor to morbidity and mortality and is in fact the leading cause of mortality in both males and females in the 25–44 years age group (8). The vast majority of doctors and nurses who serve population through a network of regional and district hospitals, health centers, and health outposts only have basic training.

Before the establishment of the University of Guyana Medical School and its 5-year Bachelor of Medicine; Bachelor of Surgery program in 1985 (9), all doctors in Guyana were trained outside the country in places such as the United Kingdom, India, Cuba, and the Soviet Union. However, it was not until the initiation of a diploma in Surgery, diploma in Orthopedics, and diploma in Nursing Anesthesia programs in 2006 at the Georgetown Public Hospital Corporation (GPHC), Guyana’s main referral and teaching hospital, that access to Graduate Medical Education became available locally. As such, with no opportunities for further training locally after internship, combined with poor income opportunity and uncertainty created by political instability, many health-care workers, in particular doctors and nurses, have migrated over the years (10). As a result, Guyana has had a shortage of health-care professionals, resulting in reduced access to basic health care in the country. The World Health Organization notes that in Guyana in 2005, there were only 2.1 physicians and 5.3 nurses and midwives per 10,000 population compared to regional figures of 20.4 and 71.5, respectively (11). Anecdotal information provided by the Medical Council of Guyana indicates that as of March 2017, there are 900 fully registered physicians and 400 institutionally registered physicians in Guyana.

Subsequent to the introduction of the above mentioned programs, several other graduate medical education programs including residencies in Emergency Medicine, Obstetrics and Gynecology, Pediatrics, Internal Medicine, and Family Practice were established at the GPHC in the 2011–2015 period, through the Institute of Health Science Education (IHSE) in collaboration with the University of Guyana and a multitude of international partners. These programs would not only provide opportunities for medical professionals to get specialist training locally but potentially be cost effective, having a holistic curriculum, teaching effective and relevant patient care skills and forming a pool of candidates to make the programs a sustainable venture.

## Beginnings of GPHC Internal Medicine/Infectious Disease (IM/ID) Residency and Journey to Graduation of First Cohort

Through a joint initiative by the IHSE, GPHC, and the Institute of Human Virology, University of Maryland and with support by a new 5-year grant from the US Centers for Disease Control and Prevention (CDC) as part of the President’s Emergency Plan for AIDS Relief, a 3-year Masters in Internal Medicine Degree program, accredited through the University of Guyana, was designed. The first batch of six residents started on January 14, 2013.

It was envisioned that the program would be supervised under the continuous presence of faculty from the University of Maryland, with the support of local faculty. The latter proved difficult with very few local IM specialists and none that were able to champion the program in leadership capacity. In August 2013, the University of Maryland hired an ID-trained physician to be the full-time in-country program director and to serve as the local champion. Somewhat unexpectedly, the funding support for the program was terminated early, creating a new challenge for the program. At this point, the program had not yet graduated its first class and had enrolled more participants. As notable gains in patient care and education had been shown, the Ministry of Health and IHSE recruited the program director to stay beyond the end of grant funding period. The primary goals during the extension were for the director to facilitate the graduation of students who would become faculty and to form new academic partnerships for curriculum development and integrity. Over the past 3 years, much progress has been made in this respect and the curriculum, in its present form, is the result of collaboration with faculty from many internationally recognized institutions of learning. This is further discussed in the sections below.

In the past more than 500-odd inpatients per month were managed by one consultant and a few general medical officers and interns, with no morning handover and rounds lasting from morning till evening. Now, a similar number of patients are managed by more than 30 doctors divided into six teams, headed, respectively, by graduates from the first cohort of students, 10 residents (3 PGY-1, 5 PGY-2, and 2 PGY-3s), several general medical officers, and interns. In terms of the resident demography, there are nine female residents and seven male residents (this includes the graduates as well), and among them nine graduated from medical school in Cuba, two in Russia, and five from the University of Guyana. The increase in the number of physicians has also been augmented by an influx of Cuban consultants hired by the Ministry of Health (who also supervise some of the teams) and large numbers of Cuban-trained Guyanese medical graduates who are returning home for further clinical training.

## The Curriculum

The stated goal of the IM residency program is to provide specialty training in IM with special focus on IDs in order to develop high-quality IM physicians who will enhance the quality of the health care and well-being of the people of Guyana. To achieve this goal, the curriculum encompasses both academic and clinical training components.

**Figure 2:**
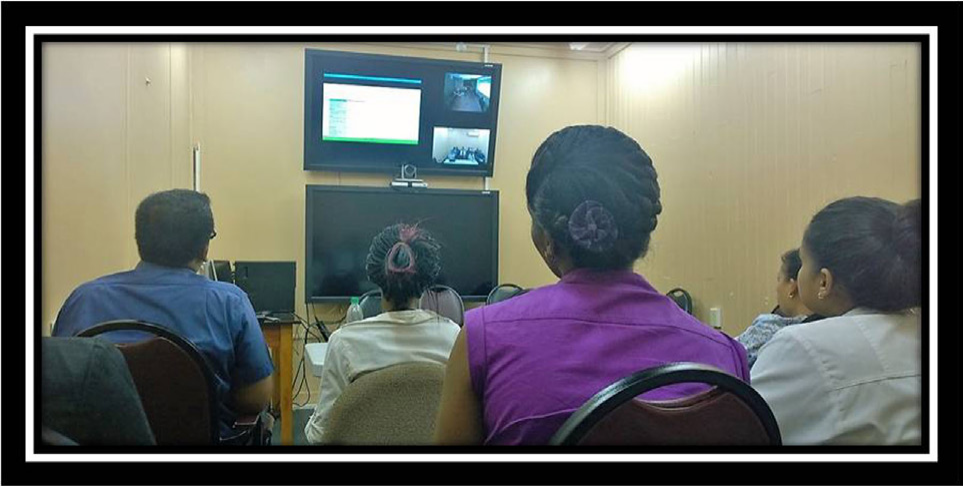
**Teleconference lectures**.

Academic activities are compulsory and are not only intended to improve the knowledge-base of residents but also to improve their scholarship, encourage the application of the best available evidence in clinical practice, and develop skills in root cause analysis and approaches to quality improvement. These activities are conducted utilizing a variety of learning modalities which include the following:

1. Modular systems-based didactic series—each module is accompanied by a study guide and it usually takes 1–2 months to complete the specific lecture series. Didactic lectures are conducted once or twice weekly either (a) in a classroom by resident or visiting faculty using slideshows, whiteboard, and bedside demonstrations where applicable or (b) via teleconference (Figure 2) with non-resident faculty or residents from other programs with whom there is collaboration. As the lecture series for each module progresses, residents simultaneously engage in a senior resident-moderated group study to cover specific objectives and practice and discuss board review questions. Each module usually concludes with a multiple choice question exam.
2. Resident case presentations—case-based discussions on diseases and their management.
3. Journal club—occurs once to twice monthly. Articles on topics of interest are chosen and reviewed with registrars and then recommended to be critically analyzed and presented in a specified format by the residents (12).
4. Morbidity and mortality rounds—these are held monthly and the medicine department meets to discuss the morbidity and mortality statistics for the previous month and two or three cases of interest. These cases are not just presented, but analyzed using a health-care matrix (13), and the points needing clarification or improvement are noted by a designated resident, who is responsible for following up for the next meeting. The points noted in these discussions also serve as starting points for potential quality improvement projects.

The clinical training component of the curriculum spans the 3 years of the program and allows residents to work and learn on rotations in a variety of clinical settings. Each rotation block usually lasts 4 weeks. The rationale behind—and expectation of residents in—these rotations are highlighted below:

1. Inpatient medicine: the inpatient IM rotations form the bulk of the rotations and serve as the foundation of the inpatient general medicine/IDs experience with an emphasis being placed on cost containment, medical ethics, and preventive medicine when applicable.

Residents are expected to function as team leaders, supervising all members of the team and guiding the clinical care of the patients and educational development of the interns and students. They also attend and guide discussions at daily morning handover, which ensures continuity of care and provides teaching opportunities. At least once a week, senior residents and registrars “run the whiteboard” and conduct hands-on resuscitation demonstrations (low fidelity simulation using resuscitation mannequins) during the morning conference.

2. ID ward: this is dedicated to the investigation and treatment of tuberculosis. Residents rotate on this service at least once per year, and this entails interacting with unique patient populations and collaborating with the National Public Health Reference Laboratory and Georgetown Chest Clinic. Students are exposed to the use of modern technologies such as GeneXpert and plan and organize long-term care for patients.
3. General cardiology and cardiac intensive care unit: this inpatient service and unit were recently established by professors from the Libin Cardiovascular Institute, University of Calgary—presently one of the major collaborators with our IM/ID residency program. Residents work at least one block a year on this service under the guidance of visiting cardiologists and fellows, and a resident registrar, being exposed to a wide range of cardiovascular pathologies in stable and critically ill patients.
4. Outpatient medicine: weekly clinics are included as part of all inpatient rotations which provide residents with an experience in outpatient management and allow them to appreciate the natural history of disease and become familiar with common problems encountered in the practice of general IM. There is a focus on preventive medicine, cost containment, and psychosocial/behavioral issues. They are also taught how and when to seek subspecialty consultation and how to provide general medicine consults to other clinical specialties.
5. HIV outpatient clinic: this rotation occurs once per year at the National Care and Treatment Institute under the guidance of its director and provides interaction with various populations including prisoners, pregnant women, members of the LGBT community and these are patients with both newly diagnosed HIV and its complications. On this rotation, residents are also exposed and involved in the diagnosis and treatment of other sexually transmitted infections.
6. Emergency room (ER): this is an intense one block rotation, the goals of which are twofold: to provide an opportunity for residents to care for patients in an acute setting with a broad spectrum of medical, surgical, and gynecological diagnoses and to expose them to a full range of acuity—from trauma and medical resuscitations to ambulatory care.
7. Intensive care unit: the intensive care unit receives patients primarily from the medical wards, ER, and transfers from other hospitals. The main goal of this rotation is to provide a milieu for residents to learn the basic principles of critical care medicine. Residents work one block per year in this unit, where they care for critically ill patients with a broad variety of medical illnesses under the guidance of a faculty member and in conjunction with the anesthesia program at GPHC.
8. Senior regional service rotations: this is a two block rotation done by senior residents at a regional hospital, such as the West Demerara Regional Hospital, in their third year of the program. The goals of this rotation are to provide the experience of more independent clinical practice, increase awareness of the systems outside of GPHC, the challenges, and advantages of practicing in such a setting. Residents are also required to provide a service to the regional hospital in either a quality improvement, educational, or research project.
9. International rotations: in collaboration with international partners from McMaster University and the University of Calgary, senior residents in their third year are able to do a two-block elective in nephrology, general IM, or cardiology at teaching hospitals associated with these institutions. International electives are an essential part of the program and not only expose residents to advanced diagnostics and treatments, and increase their depth of knowledge, but they also see what it is to practice in a highly efficient system, and inspire ideas for quality improvement back home.

In the future, there is also plan to take the residents on outreach programs to the rural areas to screen and assess for chronic medical diseases such as diabetes and hypertension and to do patient education regarding these diseases.

## Assessments

In addition to actively taking part in the academic and clinical training aspects outlined in the curriculum, residents undergo several assessments that serve a variety of purposes for both the resident and the program:

1. Clinical evaluations: residents receive clinical evaluations for each rotation based on the six-core competencies outlined by the Accreditation Council for Graduate Medical Education: Patient Care, Medical Knowledge, Practice-Based Learning and Improvement, Interpersonal and Communication Skills, Professionalism, and Systems-Based Practice (14). In addition to the assessments performed by supervisors, resident-to-resident peer assessments are also performed, which are used solely for formative feedback purposes.
2. Subject exams: at the end of a lecture series of a particular module, residents are assessed in a multiple choice question exam with case-based questions.
3. American College of Physicians (ACP) In-training exam: once a year, second- and third-year residents do the ACP In-training computer-based residency exam. Residents receive individualized reports and assessment of knowledge based on content areas for the purposes of directing their study efforts. The in-training exam is not used for purposes of promotion.
4. Annual written examination: the annual exam is written by program faculty and assesses content covered during the year. A passing score is required for promotion.
5. Final examination: at the end of the program, residents undergo a final exam to determine their competence to practice as Internists. This is both a written and oral examination. The oral portion is administered as an Objective Structured Clinical Examination designed to test communication, procedural, and clinical reasoning skills. The examination is written by program faculty in collaboration with external examiners from partners at Caribbean and/or North American Universities.

Our first cohort of six residents passed their final examinations successfully in November 2016. They started as registrars in the hospital and remain an integral part of the residency program. Since the program started, four residents left the program; one had to leave after repeatedly failing remediation courses and three left due to other personal commitments.

## Need for Collaboration

A young residency program needs a lot of nurturing to bloom to its full potential. A resource-limited setting with just one resident faculty member can lead to a challenging environment in which to make this feasible. Although GPHC had other residency programs that could mentor the medicine program—partnerships and collaborations became a necessity to develop further on the foundation of the residency program and help develop and sustain the program at international standards. Collaborations with academic leaders in the field foster a process of self-appraisal and improvement and provide networking opportunities that broaden the horizon of medical education and academic scholarship far beyond the local boundaries.

## International Collaboration

At present the GPHC program collaborates with the following universities: McMaster University, University of Calgary, University of Pittsburgh, and a recently expanded collaboration with Health Volunteer Overseas (HVO). The collaborations have been developed with an open, trusting, and negotiable memorandum of understanding. The goals of the collaboration are to focus on the following:

1. Clinical teaching and supervision

Visiting faculty from each of the universities volunteer their academic and clinical time at GPHC and are provided medical licensure by the Guyana Medical Council. They provide clinical supervision, mentorship, and bedside teaching to the residents, medical students, medical officers, and interns participating in patient care. Having experts from different specialty areas and academic backgrounds provides the learners at GPHC ongoing dynamic perspectives by which to discuss different approaches to the clinical cases that they see everyday. It also gives them an opportunity to discuss evidence-based medicine and understand the application to their local surroundings and resources.

Visiting faculty also fill in the gaps in the delivery of the formal curriculum—i.e., delivery of lectures, case discussions, and supervision of journal clubs. Didactic lectures and case-based discussions are not only facilitated in person but also *via* teleconference on a fixed schedule by faculty and residents from partner universities.

2. Curriculum development and assessment

**Figure 3:**
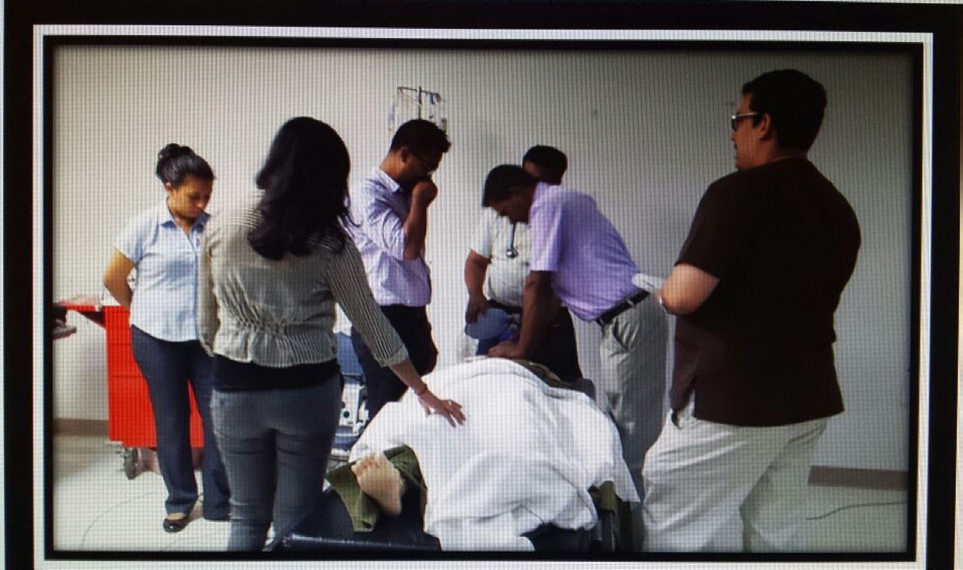
**Acute resuscitation course using simulation**.

**Figure 4:**
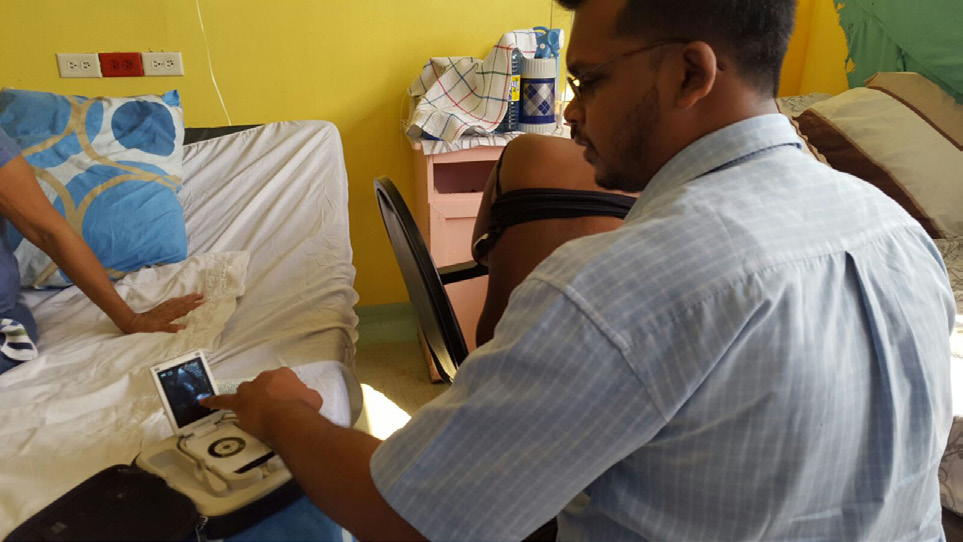
**Use of bedside ultrasound by resident**.

Although a formal curriculum has been developed by the program director and has been adapted to the local health-care needs and to the requirements of the academic program, fresh perspectives, critical appraisal, and assessment are essential. Faculty in partner universities have been recruited to collaborate on further development and delivery of the curriculum. Assessment (summative and formative) tools are being adapted and implemented to allow for more well-rounded assessment of the residents. Simulation (Figure 3) has been incorporated as part of the curriculum to teach clinical reasoning skills, resuscitation skills, and communication skills. An ultrasound curriculum (Figure 4) has been developed and implemented to assist learners in bedside clinical assessment and for improving the safety of bedside procedural skills.

Recently, 10 residents and 6 recent graduates of the IM residency program were surveyed to get their feedback on the current curriculum. This included formative feedback and summative feedback using a Likert scale (from 1 to 10 with 10 being excellent) to rate the different modalities of teaching employed in the curriculum. Ninety percentage scored the simulation-based teaching above 7, and 70% scored the didactic teaching above 7. Morbidity and mortality teaching and journal clubs were scored above 7, 80% of the time. The formative feedback included the following—the residents enjoy the simulation-based teaching as it is practical and engaging and they found they can apply the teaching to the cases that they see. As long as the didactic sessions are high quality and delivered by engaging and experienced physicians, they find the sessions relevant and informative. They did find that since the didactic sessions are done by faculty who also have to manage the patient load, some sessions were rushed. Due to the paucity of visiting faculty, it is recognized that there is a lack of diversity in subspecialty teaching. The teleconferences are useful if they are interactive and use local cases as points of discussion. The ECG teaching is repeatedly reported as being of very high quality and very applicable to their clinical practice. Although the mortality and morbidity rounds and journal clubs are scheduled regularly, the residents feel that the discussions and presentations do not always facilitate learner engagement and discussion. The point of care ultrasound was particularly identified as adding value to their clinical teaching and practice as they felt more comfortable doing bedside procedures such as thoracentesis, central line insertion, and paracentesis and are now also able to use the ultrasound for bedside assessments to help in making clinical decisions given the lack of portable chest X-rays for critically sick patients.

3. Electives

One two-block international elective in subspecialties (nephrology, general IM, or cardiology) has been organized where GPHC residents gain experience and exposure to different medical settings and practices. They are encouraged to reflect and implement some of the learned practices in their local medical environment. Residents and medical students from US and Canadian Universities are also given the opportunity to do supervised elective rotations in GPHC and participate in the academic rounds, as well as be involved in resident and medical student teaching that enriches their global health experience.

4. Research

As the residency program develops a sound foundation, research projects are being fostered with supervision and collaboration from partner universities. An online quality improvement course was taken by all the residents through the Institute of Health Improvement website, and project plans are on their way to being implemented. Small, locally applicable projects are a good way to start to garner understanding of the process and make it more feasible.

5. Mentorship and training the trainer programs

The first residents graduated in November 2016 and are now part of the local resident faculty. There is a need for ongoing mentorship as these graduates move into leadership and clinical educator roles. And collaborative partners are vital. Online sessions are organized for the graduates to meet with their mentors for debriefing and regular reflection and self-evaluation. Online and local courses are being organized for training the trainer initiatives that build on their teaching and leadership skills.

6. Resource support

In the public health-care system in Guyana (which includes teaching hospitals such as the GPHC), select essential medications and health-care services are free to all patients. Some of the collaborators have participated in improving public health care in Guyana by donating medical equipment and hard to obtain medications. Partner universities have also assisted in providing resources such as partial task trainers and mannequins for simulation training, portable ultrasound machines, learning materials, online resources, etc.

## Lessons Learned

1. The need for strong support from local leadership

While the need for specialists in IM and IDs is evident based on the number of health-care personnel in the country and the trends in the morbidity and mortality statistics, developing credible and sustainable programs to have specialist doctors trained locally is a paradigm shift in policy in Guyana. The success of such efforts is multifaceted and depends on collaboration and the continued support of a number of vital partners locally and internationally. In the case of the Guyana IM/ID residency program, the necessary local support was provided from the Ministry of Health, with favorable opinions of post-graduate medical education from the Minister and other political entities; the IHSE, which provided the platform for the program to be delivered; GPHC, which provided the clinical teaching site; and the University of Guyana, which confers the degree offered on completion of the program. As noted, the presence of a local “champion of the program” also proved to be an essential component. The local “champion” provided necessary representation to local leadership, assisted in developing bidirectional partnerships, and advocated for the specific needs of the program with international partners.

2. Diversified funding

Through engagement with multiple local and international stakeholders, funding for various aspects of the program has become diversified. The benefit of such support became evident, when the success of the program was threatened by a loss of funding from the CDC in its first year.

3. Balance and adaptation to local educational system

The curriculum was initially based on typical course work of a US-based residency program, with the addition of a focus on IDs, because of its relevance in Guyana. However, the curriculum had to be adapted within the first year for several reasons: (1) there were differences in baseline knowledge from medical school, which meant more basic science and background knowledge needed to be targeted; (2) many of the program participants had several years of clinical experience as general medical officers, so the clinical teaching needed to be targeted to a higher level; (3) similar to many resource-limited health-care settings, there are significant deficits in efficiencies and processes, so a sharper focus on system-based practice, high-quality care, and principles of quality improvement were needed in the educational program.

4. Using a variety of learning platforms

Traditional specialist training has revolved around the combined learning platforms of bedside teaching, didactic lectures, and group learning sessions. Technology is increasingly being used to diversify learning experiences and optimize use of face-to-face time between learners and experts. With the globalization of technology, programs with limited access to faculty and expertise can capitalize on the use of technology to maximize learning. The IM/ID program adopted several of these alternatives and routinely uses: teleconferencing, virtual libraries, social media, online modules for didactic teaching and interactive case discussions, and simulation-based education. These teaching modes have not only enhanced the learning experience for residents in the program, but have expanded access to expertise and clinical teaching in a way that is flexible and feasible for our international partners.

## Future Directions

1. Transition of leadership and ongoing mentorship of junior faculty

As the program produces graduates, the reins of leadership will be gradually transferred to these new locally trained specialists who will not be just clinicians but will add to the pool of “program champions” and take on the responsibility of moving the program forward. This will be another phase in the process of ensuring the program remains sustainable and is envisioned to require ongoing mentorship over the next few years. Of note, selection of residents for admission to the program, delegation of duties throughout the years of residency, implementation of resident-initiated quality improvement projects, etc. are all done in consideration of this larger picture.

2. Formal quality improvement training and collaborative QI projects based at GPHC

One of the major goals of the IM/ID residency program is to improve the standard of care for patients with conditions falling under the care of internists. While having specialists trained locally is an excellent step toward this end, implementation of best practices at a systemic level is also necessary. With all IM residents initiating various quality improvement projects during their residency years, the intellectual environment created is one that is conducive to fostering projects that will result in positive tangible changes in the delivery of cares. Having basic, formal training in quality improvement can strengthen this process and help build capacity for even larger projects.

3. Expansion of subspecialty services

As part of the collaboration with various internationally recognized institutions of learning, the possibility of having graduates of the GPHC Internal Medicine Residency pursue fellowships in subspecialties such as nephrology and cardiology is being considered. Several of the international partners have already made strides in establishing subspecialty services at the GPHC so that these ventures can be sustainable. Having a local specialist is important. This would not only be an opportunity for further professional development for the new graduates but also on a larger scale it will enhance the capacity of both the health-care and educational workforce. It will also advance the cause of improved health care for the citizens of Guyana.

4. Research collaborations

To improve both clinical and academic standards, it is vital that research should be part of the GPHC residency program. Further collaborations with partner universities are planned to encourage residents to ask, enquire, and then answer their research questions with mentorship to foster the development of adequate methodology. Ideally, the residents and medical officers at GPHC will take the lead on such research projects and then disseminate it by presenting at local and international conferences and submitting for publication in peer reviewed journals. Balancing research responsibilities with heavy clinical loads will be challenging and, therefore, priority will be given to research on quality improvement projects around medical education and patient care and safety.

5. Assessment of programs and interventions to identify what does/does not work in the setting

Ongoing external and internal, local and international assessment and appraisal of the program are vital for ongoing success, preservation, and sustainability. A memorandum of understanding outlining roles and responsibilities is vital to partnership development and enrichment of the global health curriculum and experience. Committees involving local and international stakeholders are needed to meet and review all aspects of the program. These committees will make recommendations to evolve the program and build on local and international partnerships and continue to be innovative in the field of global health.

## Conclusion

The GPHC program has come a long way since its implementation in 2013. Much of its success is attributed to the dedication and perseverance of the program director and the residents who have worked on local and international collaborations to weave a strong mesh on which to support and build this program. Barriers related to limited financial and human resources continue, and the program is working hard to be more self-sustaining and self-preserving by training the program graduates to be leaders in clinical care, education, and research. Ultimately, they will take on independent leadership of the residency program. The future definitely is not free of bumpy (yet interesting) rides but does promise new horizons for developing the program and its partnerships. The program leaders hope to move toward a competency-based design that provides a standard of assessment for graduating physicians and providing more holistic, evidence-based health care to the Guyanese population.

## Author Notes

Dr. Dev Persaud is a current third year resident in the GPHC IM/ID residency program. Dr. Joanna Cole is the current program director for the IM/ID residency program and works as an Infectious Disease specialist at V.A. Medical Centre, Spokane, WA. Dr. Ramdeo Jainarine is a 2016 graduate of the IM/ID program and now the assistant program director for the residency program and the on site program director for the HVO volunteer program. Dr. Zahira Khalid is associate professor at McMaster University, Volunteer Assistant Program Director for the GPHC IM/ID residency program and external site director for the HVO volunteer program.

## Author Contributions

DP is the first author and contributed to 40% of the writing and gathering of the information for the article. JC is the second author and contributed to 30% of the material in the article. RJ contributed to 10% of the article in terms of material and writing. ZK supervised and mentored DP in the writing and editing of this manuscript and contributed to 20% of the material in the script.

## Conflict of Interest Statement

The authors declare that the research was conducted in the absence of any commercial or financial relationships that could be construed as a potential conflict of interest.
